# Risk of venous thromboembolism associated with Janus kinase inhibitors for rheumatoid arthritis: case presentation and literature review

**DOI:** 10.1007/s10067-021-05911-4

**Published:** 2021-09-23

**Authors:** Shunsuke Mori, Fumihiko Ogata, Ryusuke Tsunoda

**Affiliations:** 1 Department of Rheumatology, Clinical Research Center for Rheumatic Diseases, National Hospital Organization Kumamoto Saishun Medical Center, 2659 Suya, Kohshi, Kumamoto 861-1196 Japan; 2 Divsion of Cardiology, Japanese Red Cross Kumamoto Hospital, Kumamoto, Japan

**Keywords:** Baricitinib, Deep vein thrombosis, Janus kinase inhibitors, Pulmonary embolism, Rheumatoid arthritis, Tofacitinib

## Abstract

Janus kinase (JAK) inhibitors have been developed as disease-modifying antirheumatic drugs. Despite the positive therapeutic impacts of JAK inhibitors, concerns have been raised regarding the risk of venous thromboembolism (VTE), such as deep vein thrombosis (DVT) and pulmonary embolism (PE). A recent post hoc safety analysis of placebo-controlled trials of JAK inhibitors in rheumatoid arthritis (RA) reported an imbalance in the incidence of VTE for a 4-mg daily dose of baricitinib versus placebo. In a recent postmarketing surveillance trial for RA, a significantly higher incidence of PE was reported in treatment with tofacitinib (10 mg twice daily) compared with tofacitinib 5 mg or tumor necrosis factor inhibitors. We also experienced a case of massive PE occurring 3 months after starting baricitinib (4 mg once daily) for multiple biologic-resistant RA. Nevertheless, the evidence to support the role of JAK inhibitors in VTE risk remains insufficient. There are a number of predisposing conditions and risk factors for VTE. In addition to the known risk factors that can provoke VTE, advanced age, obesity, diabetes mellitus, hypertension, hyperlipidemia, and smoking can also contribute to its development. Greater VTE risk is noted in patients with chronic inflammatory conditions, particularly RA patients with uncontrolled disease activity and any comorbidity. Prior to the initiation of JAK inhibitors, clinicians should consider both the number and strength of VTE risk factors for each patient. In addition, clinicians should advise patients to seek prompt medical help if they develop clinical signs and symptoms that suggest VTE/PE.

**Table:** 

**Key Points** *• Patients with rheumatoid arthritis (RA) are at increased risk of venous thromboembolism (VTE), especially those with uncontrolled, high disease activity and those with comorbidities.* *• In addition to the well-known risk factors that provoke VTE events, advanced age and cardiovascular risk factors, such as obesity, diabetes mellitus, hypertension, hyperlipidemia, and smoking, should be considered risk factors for VTE.* *• Although a signal of VTE/pulmonary embolism (PE) risk with JAK inhibitors has been noted in RA patients who are already at high risk, the evidence is currently insufficient to support the increased risk of VTE during RA treatment with JAK inhibitors.* *• If there are no suitable alternatives, clinicians should prescribe JAK inhibitors with caution, considering both the strength of individual risk factors and the cumulative weight of all risk factors for each patient.*

**Key Points**

*• Patients with rheumatoid arthritis (RA) are at increased risk of venous thromboembolism (VTE), especially those with uncontrolled, high disease activity and those with comorbidities.*

*• In addition to the well-known risk factors that provoke VTE events, advanced age and cardiovascular risk factors, such as obesity, diabetes mellitus, hypertension, hyperlipidemia, and smoking, should be considered risk factors for VTE.*

*• Although a signal of VTE/pulmonary embolism (PE) risk with JAK inhibitors has been noted in RA patients who are already at high risk, the evidence is currently insufficient to support the increased risk of VTE during RA treatment with JAK inhibitors.*

*• If there are no suitable alternatives, clinicians should prescribe JAK inhibitors with caution, considering both the strength of individual risk factors and the cumulative weight of all risk factors for each patient.*

## Introduction

The Janus kinase (JAK)/signal transducer and activator of transcription (STAT) pathway is one of the major cascades that transfers extracellular cytokine signals from cell surface receptors to the nucleus. There are four isoforms in the JAK family, namely, JAK1, JAK2, JAK3, and TYK2, which act in pairs either as homodimers or as heterodimers to activate STAT proteins. Different cytokine receptor families utilize specific pairs of JAK isoforms for signal transduction [1, 2].

Over the last decade, JAK inhibitors, small molecules that target the JAK-STAT signaling pathway, have been developed as targeted synthetic disease–modifying antirheumatic drugs (tsDMARDs) for immune-mediated inflammatory diseases (IMIDs) such as rheumatoid arthritis (RA) [3–5]. Biological DMARDs (bDMARDs), protein molecules that target specific cytokines and cytokine receptors in the inflammatory cascade, have several limitations, including the need for parenteral administration and the development of anti-drug antibodies due to inherent immunogenicity [6]. In the context of these limitations, JAK inhibitors have significant advantages over bDMARDs. In addition, recent randomized clinical trials of JAK inhibitors for RA demonstrated equivalent or even superior efficacy to adalimumab, a tumor necrosis factor (TNF) inhibitor [7–10]. Using real-world registries, we showed that tofacitinib, a first-generation JAK inhibitor, can induce greater improvements during the first 12-month treatment in bDMARD-naïve RA patients compared with tocilizumab, an anti-interleukin-6 receptor antibody [11, 12]. Despite these positive therapeutic impacts of JAK inhibitors, concerns have been raised regarding the risk of venous thromboembolism (VTE), such as deep vein thrombosis (DVT) and pulmonary embolism (PE). In addition, previous meta-analyses indicated a higher background risk of VTE among patients with RA or other IMIDs compared with the general population [13, 14].

The aim of this review is to provide the latest update regarding the risk of VTE events associated with JAK inhibitors in RA patients, which can guide therapeutic decisions based on safety considerations. We also share our recent experience with a case of massive PE occurring in the treatment of multiple biologic-resistant RA with a JAK inhibitor, baricitinib, with the intention to discuss the risk management of VTE events.

## Case presentation: massive PE during baricitinib therapy for RA

In April 2010, a 46-year-old female was diagnosed with seropositive RA. The disease activity was moderate. The patient started methotrexate (MTX) monotherapy, but it failed to control the disease activity. Next, the patient attempted four different biological therapies sequentially, starting with etanercept plus MTX, then proceeding to infliximab plus MTX, tocilizumab plus MTX, and abatacept monotherapy, but every therapy failed and the disease activity became high. In March 2020, high-throughput leukocytapheresis (LCAP), which is an alternative therapeutic option for the management of RA with super-resistance to DMARD therapies [15], was initiated. After five LCAP procedures at 1-week intervals, the patient started baricitinib, a JAK1/JAK2 inhibitor, 4 mg once daily with oral prednisolone. Eight weeks later, the patient achieved low disease activity. Twelve weeks after starting baricitinib therapy, dyspnea and chest pain suddenly appeared on lifting heavy objects. The patient had noticed painless swelling of the left leg 1 week prior to this attack. The patient was immediately taken to an emergency hospital by ambulance because of worsening dyspnea.

**Fig. 1:**
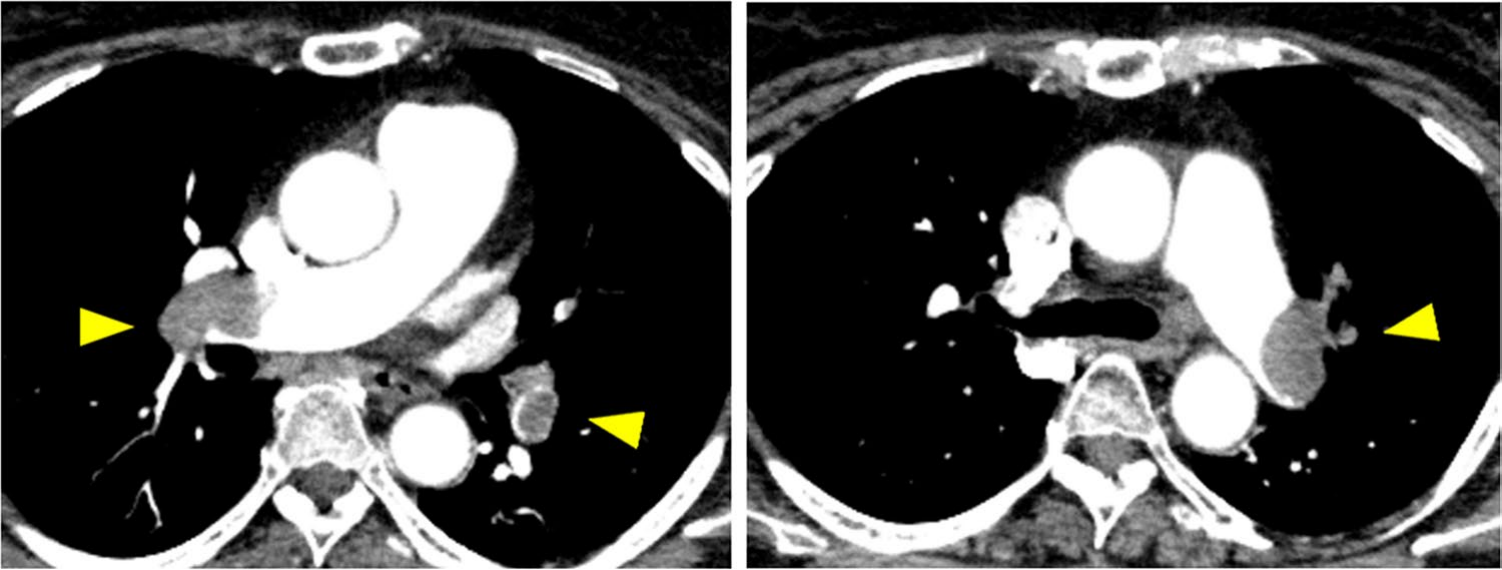
Contrast-enhanced computed tomography reveals prominent emboli in the bilateral main pulmonary arteries (yellow arrowheads)

**Fig. 2:**
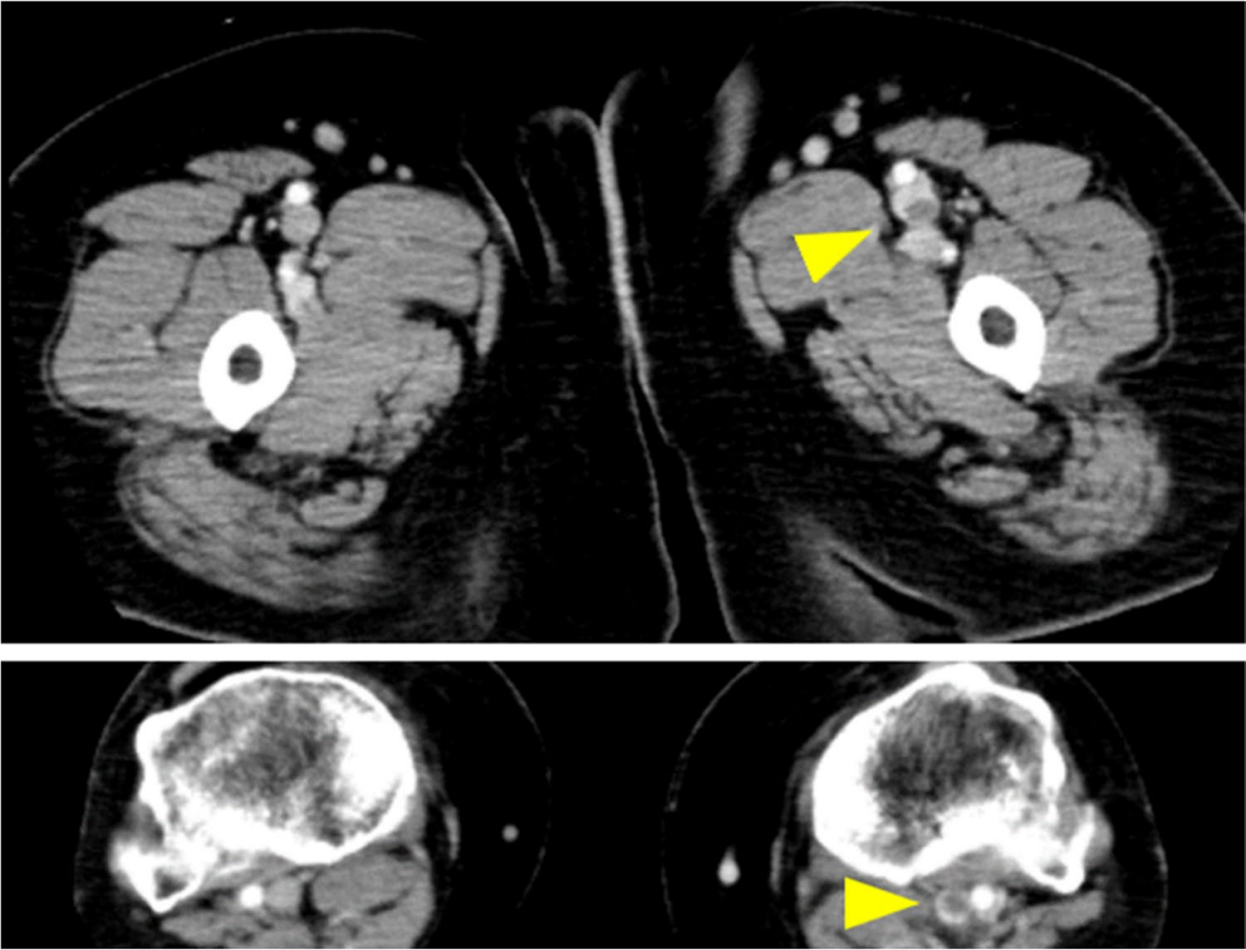
Contrast-enhanced computed tomography reveals occlusive intravenous thrombosis in the left popliteal vein and the left superficial femoral vein (yellow arrowheads)

In the emergency room, the patient was in shock. The respiratory rate was 30 breaths/min and SpO_2_ was 90% with reservoir mask oxygen at 7 L/min. Arterial blood gas analysis showed PaO_2_ of 77 Torr, PaCO_2_ of 29 Torr, and HCO_3_– of 19.2 mmol/L. Elevated levels of serum D-dimer (34.6 µg/mL) and brain natriuretic peptide (BNP, 30.1 pg/mL) were observed. The electrocardiogram indicated right ventricular strain with a heart rate of 126 beats/min. Transthoracic echocardiography showed a dilated right ventricular dimension (50.5 mm), McConnell sign (defined as right ventricular free wall akinesis with sparing of the apex), and reduced tricuspid annular plane systolic excursion (TAPSE) to 9.3 mm. These results indicate severe right ventricular systolic dysfunction. Contrast-enhanced computed tomography revealed thrombi in both main pulmonary arteries, the left popliteal vein, and the left superficial femoral vein (Figs. 1 and 2). The patient was diagnosed as developing acute massive PE caused by DVT [16–18]. Anti-phospholipid syndrome–related tests and anti-SARS-Cov-2 antibody tests were negative. The body mass index was 34.2 (obese class I), and no other cardiovascular or VTE risk factors were identified.

The patient was intravenously administered 120 × 10^4^ units of tissue-type plasminogen activator (t-PA) as thrombolytic therapy. On admission day 2, the patient recovered from the shock state, and dyspnea was improved. No bleeding was observed. Oral rivaroxaban 30 mg daily (Xa inhibitor) was used as anticoagulation therapy. On admission day 6, the patient’s dyspnea and hypoxia were resolved. Contrast-enhanced computed tomography revealed that the amounts of thrombi had decreased. The findings of right ventricular strain disappeared. On admission day 10, the patient was discharged with oral rivaroxaban. Certolizumab-pegol plus MTX therapy was newly started. Four months later, the patient achieved low disease activity, and the emboli disappeared from the pulmonary arteries and the veins of the left lower limb.

The latest postmarketing surveillance data on safety from pharmaceutical companies in Japan reported six cases of DVT (0.09%), two cases of PE (0.03%), and one case of venous embolism (0.01%) in RA patients receiving tofacitinib (*n* = 6989, data cutoff May 5, 2020), and 11 cases of severe VTE (0.3%) and seven cases of non-severe VTE (0.2%) in RA patients receiving baricitinib (*n* = 3445, data cutoff January 1, 2021). In our institution, tofacitinib or baricitinib was used in approximately 200 RA patients and, as mentioned above, one patient developed massive PE 3 months after starting baricitinib 4 mg once daily.

## Search strategy

The literature search for the current review was carried out in line with the recommendations for bibliographic searches for narrative reviews [19]. Using the PubMed platform, the Medline database was searched on April 30, 2020, for English biomedical literature focusing on VTE risk in RA patients receiving and not receiving JAK inhibitors. The identification of eligible articles was initially carried out by screening titles and abstracts, and finally by reading the full text of the publication. The references of the eligible articles were screened to ensure that no important research data relevant to the subject were missed.

To identify English articles relating to the VTE risk associated with JAK inhibitors, we used the terms (venous thromboembolism OR venous thromboembolic event OR pulmonary embolism OR deep vein thrombosis) AND (Janus kinase inhibitor OR tofacitinib OR baricitinib OR upadacitinib OR filgotinib OR peficitinib). Through the Medline search, a total of 90 articles were identified. Among them, we found eight post hoc safety analyses, two systematic reviews, and seven systematic reviews/meta-analyses using pooled data from clinical trials and long-term extension (LTE) studies of JAK inhibitors for RA and other IMIDs. In addition, six postmarketing studies using real-world registries of RA and other IMID patients receiving JAK inhibitors were identified (among these 6, one study was also identified and included as a post hoc analysis). We also found three review articles including detailed data on incidence rates of VTEs associated with JAK inhibitors. All of these studies are included in the “VTE events in RA patients receiving JAK inhibitors” section of this review. Individual clinical trials as well as LTE studies were not included in this section because all VTE cases in these studies were incorporated into the abovementioned post hoc analyses and systematic reviews/meta-analyses. We also excluded studies that only focused on patients with non-RA IMIDs.

To identify English articles comparing VTE risk between RA patients and non-RA controls as well as those comparing VTE risks among RA patients based on disease activity, we used the terms (venous thromboembolism OR venous thromboembolic event OR pulmonary embolism OR deep vein thrombosis) AND (rheumatoid arthritis OR autoimmune OR immune-mediated OR inflammatory) AND (incidence OR rate). Through the Medline search, a total of 1608 English articles were identified. Among them, we found 16 eligible articles (15 articles comparing the VTE incidence between RA patients and non-RA controls and one article comparing the VTE incidence among RA patients based on disease activity/severity). These studies are included in the “VTE risks in RA patients” section of this review. Studies focusing on postoperative VTE events or recurrent VTE events were excluded.

Additional articles were also selected based on the prior knowledge of the authors, and the information was synthesized below.

## A brief overview of VTE

### Epidemiology of VTE

VTE is fairly common, and its incidence increases exponentially with age [20, 21]. In the majority of cases, VTE manifests as DVT of the legs and pelvis; in 30 to 40% of patients, it appears as PE. The estimated annual incidence rates (IRs) for VTE, PE (with or without DVT), and DVT alone in Western countries are reported to range from 104 to 183, 29 to 78, and 45 to 117 per 100,000 person-years, respectively. The recurrence of VTE occurs frequently: approximately 30% of patients who have a first episode of VTE will experience recurrence within 10 years [22]. A VTE event is a serious medical condition associated with long-term morbidity and increased mortality. In particular, PE is an independent predictor of reduced survival for up to 3 months after the event [23, 24]. As a result of the westernized lifestyle and aging society in Japan, the number of patients with PE has increased 4.6-fold in the past 15 years, with an estimated annual IR of 12.6 per 100,000 person-years in 2011 [25].

### Risk factors for VTE

In the nineteenth century, the German physician Virchow explained the pathophysiologic mechanisms of VTE by three major determinants, called Virchow’s triad, which included hypercoagulability (constituents of blood), endothelial injury (vessel wall), and venous stasis (blood flow) [26]. There are a number of predisposing conditions and risk factors for VTE, which can affect one or more elements of Virchow’s triad.

Many episodes of VTE are provoked by a transient or persistent risk factor [27]. Major general surgery, orthopedic surgery (hip or knee replacement), major trauma, fracture (hip or leg), spinal cord injury, and immobilization are categorized as major setting-related (usually transient) risk factors that can provoke VTE. Active cancer is a well-recognized patient-related (usually permanent or progressive) risk factor that can provoke VTE [18, 20, 27, 28]. In addition, congestive heart or respiratory failure, infection (such as pneumonia, urinary tract infection, or HIV infection), and acquired thrombophilia (antiphospholipid antibody syndrome, etc.) are considered moderate-risk factors that can provoke VTE. Heart disease such as myocardial infraction and atrial fibrillation (within the previous 3 months) especially increases the risk of PE. In women, pregnancy and puerperium, oral contraceptives, and hormone replacement therapy are recognized as moderately provoking risk factors for VTE [18, 20, 27–29]. A high risk of recurrence has been noted in patients with persistent risk factor(s). A previous episode of VTE should be considered a major risk factor for a new episode [18, 20, 22, 27].

Approximately 40 to 50% of VTE cases are considered unprovoked or idiopathic, that is, they do not have important provoking factors for VTE (either transient or persistent) [21, 27, 30]. These patients may, however, have minor acquired or inherited predisposing conditions for VTE [25, 27, 30]. Hereditary thrombophilia (antithrombin, protein C, or protein S deficiency, Factor V Leiden or prothrombin G20210A gene mutation, etc.) is considered a minor inherited risk factor. Increasing age is also associated with the risk of VTE [20, 27, 30]. Recently, the contribution of non-cancer persistent conditions, including chronic inflammatory diseases and traditional cardiovascular risk factors (such as smoking, obesity, hypertension, diabetes mellitus, and hyperlipidemia) to the pathophysiology of VTE, has been investigated. These conditions may be insufficient to cause VTE when isolated, but they can be factors that predispose an individual to VTE if combined [30].

It is becoming clear that there is a functional interdependence between inflammation and thrombosis, which is mediated by the loss of normal functions of endothelial cells, leading to the dysregulation of coagulation, platelet activation, and leukocyte recruitment in the microvasculature. Chronic inflammation appears to be an important determinant of chronic VTE events [30–32]. An imbalance between pro-thrombotic and anti-thrombotic cytokines may be involved in the pathophysiology of VTE [32].

## VTE risks in RA patients

A number of population-based epidemiological studies showed that the risk of VTE is increased in RA patients compared with the general population. Fifteen studies are summarized in Table 1 [33–47]. RA patients were more likely to experience VTE compared with age- and sex-matched non-RA subjects, even after adjustment for VTE risk factors and comorbidities. In several studies, the VTE risk was stable over follow-up time [36, 39]. In other studies, the VTE risk was highest during the first year, then attenuated with time but remained statistically elevated even 5 years after RA diagnosis [42, 46]. Among hospitalized RA patients, the PE risk was highest during the first year after hospitalization. This risk decreased over time but persisted up to 10 years [41]. These findings suggested that RA should be regarded as a hypercoagulable disorder.

**Table 1:** VTE risks in RA patients versus non-RA controls

Study	Period	Database	No. of VTEs/total RA patients	No. of VTEs/total controls	HRs/RRs/ORs/SIR (95% CI)*	Comments
	(Mean follow-up)					
	Country					
Bacani et al. [33]	1995–2008	Olmsted County, Minnesota	VTE 19/464	7/464	HR 3.6 (1.5–8.6)	Incident RA patients Matched controls (1:1)
	(5.9 years)		PE 12/464	5/464	–	
	US		DVT 11/464	4/464	–	
Matta et al. [34]	1979–2005	NHDS	VTE 110,000	10,226,000	RR 1.99 (1.98–2.00)	Hospitalized patients without joint surgery
	(NA)		PE 41,000	3,366,000	RR 2.25 (2.23–2.27)	
	US		DVT 79,000	7,681,000	RR 1.90 (1.89–1.92)	
			/4,818,000	/891,055,000		
Kim et al. [35]	2001–2008	US insurance claims database	VTE 265/22,143	448/88,572	HR 1.4 (1.1–1.7)	Matched controls (1:4) Adjusted for risk factors
	(2.0 years)		PE 111/22,143	164/88,572	HR 1.9 (1.3–2.7)	
	US		DVT 197/22,143	364/88,572	HR 1.2 (0.9–1.5)	
Yusuf et al. [36]	2007–2010	Truven Health MarketScan database	VTE 909/70,768	981/198,044	HR 2.13 (1.89–2.40) at 1 year HR 2.03 (1.64–2.51) at 4 years	Adjusted for age, sex, and risk factors
	(2.6 years)					
	US					
Bleau et al. [37]	2003–2011	HCUP-NIS database	VTE 9/5780	5716/7,917,453	OR 1.95 (1.01–3.75)	Pregnant women Adjusted for age
	(cross-sectional)		PE 5/5780	1734/7,917,453	OR 3.62 (1.50–8.70)	
	US		DVT 6/5780	4228/7,917,453	OR 1.75 (0.78–3.89)	
Yusuf et al. [38]	2010	HCUP-NIS database	VTE 2.65%/94,585	2.28%/5,539,809	OR 1.17 (1.13–1.21)	Hospitalized patients Adjusted for age, sex, race, and risk factors
	(cross-sectional)					
	US					
Holmqvist et al. [39]	1997–2010	SRQ Register	VTE 223/7904	648/37,350	HR 1.6 (1.4–1.9) HR 1.6 (1.1–2.5) within the first year HR 1.9 (1.4–2.4) at 5–9 years	Incident RA Matched controls (1:5) Adjusted for age
	(5.8 years, median)					
	Sweden					
Molander et al. [40]	2006–2018	SRQ Register	VTE 2241/46,316	5301/215,843	RR 1.88 (1.65–2.15) within the first year RR 2.03 (1.73–2.38) for high DAS28 vs. remission	Matched controls (1:5) Adjusted for age, sex, and calendar year of visit
	(1 year)					
	Sweden					
Zoller et al. [41]	1964–2008	MigMed2 database	PE 2500/86,366	–	SIR 1.91 (1.83–1.98) SIR 5.99 (5.59–6.41) within the first year SIR 1.18 (1.06–1.31) at 5–10 years	Hospitalized patients Adjusted for age, sex, entry time, and risk factors
	(NA)					
	Sweden					
Choi et al. [42]	1986–2010	THIN	VTE 176/9589	815/95,776	RR 2.14 (1.80–2.54)	Incident RA Matched controls (1:10) Adjusted for risk factors RRs remained high at ≥ 5 years^†^
	(5.5 years)		PE 82/9589	358/95,776	RR 2.16 (1.68–2.79)	
	UK		DVT 110/9589	512/95,776	RR 2.16 (1.74–2.69)	
Ogdie et al. [43]	1994–2014	THIN	(Without DMARD)			Matched controls Adjusted for age, sex, and risk factors
	(No DMARD: 5.8 years)		VTE 851/20,426	30,356/1,225,571	HR 1.29 (1.18–1.39)	
			PE 186/20,426	6066/1,225,571	HR 1.45 (1.23–1.72)	
	(With DMARD: 6.2 years)		DVT 702/20,426	25,490/1,225,571	HR 1.25 (1.15–1.37)	
			(With DMARD)			
	UK		VTE 1479/31,336	30,356/1,225,571	HR 1.35 (1.27–1.44)	
			PE 393/31,336	6066/1,225,571	HR 1.74 (1.55–1.99)	
			DVT 1162/31,336	25,490/1,225,571	HR 1.29 (1.20–1.38)	
Galloway et al. [44]	1999–2019	UK RCGP-RSC database	VTE 845/23,410	2020/93,640	HR 1.54 (1.40–1.69)	Matched controls (1:4) Adjusted for age, sex, race, and risk factors
	(8.2 years)		PE 373/23,408	916/93,639	HR 1.57 (1.36–1.80)	
	UK		DVT 542/23,408	1242/93,640	HR 1.64 (1.45–1.84)	
Ramagopalan et al. [45]	1999–2008	English national HES	6825/268,005	–	Rate ratio for VTE 1.75 (1.70–1.80)	Hospitalized patients Adjusted for age, sex, time period, and residential area
	(NA)					
	UK					
Li et al. [46]	1997–2009	British Columbia	VTE 1432/39,142	2059/78,078	HR 1.28 (1.20–1.36)	Incident RA Matched controls (1:2) Adjusted for age, sex, and risk factors HRs remained high within the first 5 years^‡^
	(9.7 years)		PE 543/39,142	791/78,078	HR 1.25 (1.13–1.39)	
	Canada		DVT 1068/39,142	1484/78,078	HR 1.30 (1.21–1.40)	
Chung et al. [47]	1998–2010	Taiwan NHIRD	PE 70/29,238	139/116,952	HR 2.07 (1.55–2.76)	Matched controls (1:4) Adjusted for age, sex, and risk factors
	(6.6 years)		DVT 208/29,238	255/116,952	HR 3.36 (2.79–4.03)	
	Taiwan					

VTE events included PE and DVT, occurring both individually and in combination

^*^The HR, RR, and OR of VTE events in RA patients were calculated compared with those in non-RA patients. Factors used for adjustment are described in the “Comments” column. The SIR was calculated by dividing the observed number of VTE cases in the RA group by the expected number of cases in the reference population with the indirect standardization method. The rate ratio was calculated as the ratio of the observed/expected numbers in the RA cohort to those in the reference cohort

^†^The time-specific RRs were highest within the first year after RA diagnosis (3.27 [95% CI 1.78–6.00] for PE and 3.16 [95% CI 1.95–5.11] for DVT), but persisted at elevated levels at 5 years and more (2.35 [95% CI 1.59–3.46] for PE and 2.32 [95% CI 1.64–3.27] for DVT)

^‡^The time-specific HRs were highest during the first year after RA diagnosis (1.60 [95% CI 1.27–2.00] for VTE, 1.86 [95% CI 1.21–2.86] for PE, and 1.59 [95% CI 1.20–2.10] for DVT), but persisted at high levels within the first 5 years (1.28 [95% CI 1.15–1.42] for VTE, 1.29 [95% CI 1.09–1.53] for PE, and 1.27 [95% CI 1.12–1.43] for DVT)

*RA*, rheumatoid arthritis; *VTE*, venous thromboembolism; *PE*, pulmonary embolism; *DVT*, deep vein thrombosis; *HR*, hazard ratio; *RR*, risk ratio; *OR*, odds ratio; *SIR*, standardized incidence ratio; *DAS28*, disease activity score for 28 joints; *NHDS*, National Hospital Discharge Survey; *HCUP-NIS*, Health Care Cost and Utilization Project National Impatient Sample; *SRQ*, Swedish Rheumatology Quality; *THIN*, The Health Improvement Network; *RCGP-RSC*, Royal College General Practitioners Research and Surveillance Center; *HES*, Hospital Episode Statistics; *NHIRD*, National Health Insurance Research Database; *NA*, not available

The VTE risk increased with increased disease activity: a twofold increase in VTE risk was observed in RA patients with high disease activity compared with patients in remission (risk ratio [RR] 2.03, 95% confidence interval [CI] 1.73–2.38) [40]. Poorly controlled RA activity may be associated with the risk of VTE. Using the Optum Clinformatics Data Mart, a United States (US) claims database that includes patients receiving DMARD treatment after the first diagnosis of RA between 2007 and 2017, Liang et al. showed that, after adjustment for multiple risk factors, patients who switched from a bDMARD/tsDMARD to another bDMARD/tsDMARD (bDMARD/tsDMARD switchers) had an increased risk of VTE compared with conventional synthetic DMARD (csDMARD) users (adjusted hazard ratio [HR] 1.36, 95% CI 1.16–1.58). Compared with first bDMARD/tsDMARD users, the adjusted HR (95% CI) for VTE was 1.35 (1.15–1.60) for first bDMARD/tsDMARD switchers and 1.48 (1.19–1.85) for second bDMARD/tsDMARD switchers. These findings suggested that switching bDMARD/tsDMARD may be a proxy for higher disease severity and poorly controlled disease activity in RA [48]. The increased VTE risk observed in RA patients may be attributed, at least in part, to uncontrolled disease activity.

## JAK inhibitors currently licensed for RA treatment

Tofacitinib and baricitinib are first-generation JAK inhibitors, and both have been approved by the US Food and Drug Administration (FDA) and the European Medicines Agency (EMA) [49, 50]. Tofacitinib, a JAK1, JAK2, and JAK3 pan-inhibitor, was first approved for the treatment of moderately to severely active RA by the FDA in 2012. In 2017, the EMA also recommended the approval of tofacitinib for RA. Currently, the recommended dose of tofacitinib in RA treatment is 5 mg twice daily in most countries. Baricitinib, which has a specificity for JAK 1 and JAK2, is the second approved JAK inhibitor. The use of this drug was approved by the EMA in 2017 at 2 mg or 4 mg once daily for the treatment of moderately to severely active RA. Subsequently, the FDA recommended the approval of a baricitinib 2-mg once-daily dosing regimen for RA treatment in April 2018, but did not recommend the use of 4 mg once daily due to safety concerns related to VTE. In Japan, baricitinib is available in 2 mg and 4 mg once-daily dosing regimens for the treatment of RA.

The next-generation JAK inhibitors upadacitinib and filgotinib were designed with selective affinity to JAK1, which may decrease the risk of unwanted adverse events without compromising clinical efficacy. Upadacitinib was approved by the FDA and EMA for the treatment of moderate to severe RA in 2019. Filgotinib was approved by the EMA, but the FDA did not approve this drug because of concerns relating to its testicular toxicity [50, 51].

These four JAK inhibitors are currently available in the treatment of RA in Japan. Peficitinib, a pan JAK inhibitor (a JAK1, JAK2, and JAK 3 inhibitor), is also approved in Japan [50].

## VTE events in RA patients receiving JAK inhibitors

### Are JAK inhibitors associated with an increased risk of VTE?

Numerically higher rates of VTE/PE events were observed in some clinical trials of JAK inhibitors versus placebo, suggesting an increased risk for developing VTE during treatment with JAK inhibitors [5, 52]. Given the rarity of VTE events, however, it is difficult to identify statistically clear signals for increased VTE risks in individual clinical trials. In addition, the higher background thromboembolic risk in RA patients versus non-RA patients may make it complicated to confirm or exclude a significant difference in risk between JAK inhibitors and placebo [53, 54]. To address this issue, a number of post hoc safety analyses and systematic reviews/meta-analyses of clinical trials and LTE studies as well as postmarketing studies using real-world registries have been conducted.

### Post hoc safety analyses of VTE events in clinical trials and LTE studies

There are eight post hoc safety analyses for clinical trials and LTE studies of four JAK inhibitors, namely, tofacitinib, baricitinib, upadacitinib, and peficitinib, for RA [55–62].

#### Baricitinib

In post hoc safety analyses using integrated data pooled from phase I, II, and III clinical trials (8 studies) as well as one LTE study of baricitinib for RA, no VTE events occurred in 1070 placebo-treated patients, but six VTE events were observed in 997 patients treated with a 4-mg daily dose of baricitinib during the 24-week placebo-controlled period. All VTE patients had conventional VTE risk factors. During extended observations, the IRs were similar between baricitinib 2 and 4 mg, with IRs of 0.5 per 100 patient-years versus 0.6 per 100 patient-years. In all patients receiving baricitinib (All-Bari-RA, a total of 3492), the IR was 0.5 per 100 patient-years and stable over time [55, 56]. The IR of VTE events increased with older age in the All-Bari-RA group [63]. In post hoc safety analyses that were limited to Japanese or East Asian patients in the ALL-Bari-RA group (5 phase II and III trials and 1 LTE study), the IRs of DVT were 0.3 to 0.5 per 100 patient-years and there were no PE events [57, 58].

#### Tofacitinib

In a post hoc safety analysis of pooled data from phase I, II, III, and IIIb/IV clinical trials as well as LTE studies of tofacitinib for RA (a total of 7964 tofacitinib-treated patients), the IRs of thromboembolic events (per 100 patient-years) in patients receiving tofacitinib 5 mg and 10 mg twice daily were 0.17 and 0.15 for DVT, 0.12 and 0.13 for PE, and 0.24 and 0.26 for VTE, respectively. The IRs in patients with and without cardiovascular risk factors were 0.24 and 0.11 for DVT, 0.25 and 0.06 for PE, and 0.43 and 0.15 for VTE, respectively. The IRs in patients with and without VTE risk factors were 0.21 and 0.07 for DVT, 0.16 and 0.04 for PE, and 0.35 and 0.10 for VTE, respectively. Thus, the IRs of VTE events in the tofacitinib development program were similar between 5 and 10 mg twice-daily doses, and higher in patients with cardiovascular or VTE risk factors versus those without. Similar findings were obtained in patients with psoriatic arthritis and those with psoriasis [59]. Similar IRs were obtained from another integrated safety analysis of data from phase I, II, III, and IIIb/IV clinical trials (19 studies), and LTE studies (2 studies) of tofacitinib for RA (a total of 7061 tofacitinib-treated patients) [60].

#### Upadacitinib

In a post hoc safety analysis using integrated data pooled from phase III clinical trials (5 studies) of upadacitinib for RA (a total of 3834 upadacitinib-treated patients), the IRs of VTE events (per 100 patient-years) in patients receiving upadacitinib 15 mg and 30 mg once daily were 0.6 and 0.3, respectively. The IRs were similar across treatment groups (0.4 for placebo, 0.5 for MTX, and 1.1 for adalimumab) [61].

#### Peficitinib

In a post hoc pooled safety analysis using integrated data from phase IIb and III clinical trials (3 trials) as well as one LTE study of peficitinib for RA (a total of 1052 peficitinib-treated patients), the IR of VTE events was 0.1 per 100 patient-years for peficitinib-treated patients, and no VTE events were observed in the placebo group. No dose-dependency was observed [62].

### Systematic reviews/meta-analyses of clinical trials and LTE studies

Seven meta-analyses using data extracted from clinical trials of JAK inhibitors for RA and other IMIDs were identified in the literature. These studies are summarized in Table 2 [64–70]. The meta-analyses for RA showed that there was no significant difference in the risk of VTE events between patients receiving JAK inhibitors and those receiving placebo. During the limited placebo-controlled periods, no dose-dependent impact on the risk of VTE events was observed in tofacitinib (5 mg vs. 10 mg twice daily), baricitinib (2 mg vs. 4 mg once daily), or upadacitinib (15 mg vs. 30 mg once daily) [64, 65]. The meta-analyses for IMIDs (including RA) showed that VTE risk was unlikely to substantially increase in patients receiving JAK inhibitor during the limited placebo-controlled periods [66–69]. In a stratified and meta-regression analysis, there was no interaction by dose of JAK inhibitors, indication for treatment, or length of follow-up [68]. In an indirect meta-analysis, the risk of VTE events in tofacitinib-treated patients was lower than in baricitinib-treated patients (OR 0.09, 95% CI 0.02–0.51), suggesting the superior safety profile of tofacitinib to baricitinib [69]. No increased risk was found for PE during treatment with JAK inhibitors for IMIDs including RA [70].

**Table 2:** Meta-analyses of VTE risk in clinical trials of JAK inhibitors for RA and other IMIDs

Study	JAK inhibitors	No. of study^†^	JAK inhibitors^†^		Placebo^†^		ORs/RRs/RDs (95% CI) *	Others
			Events	Total	Events	Total		
Xie et al. [64]	Overall	25 for RA	12	2193 PYs	3	982 PYs	OR 1.16 (0.48–2.81)	(Dose dependency: OR)
	Tofacitinib	9	1	809 PYs	2	205 PYs	OR 0.17 (0.03–1.05)	5 vs. 10 mg: 0.81 (0.22–3.03)
	Baricitinib	6	7	693 PYs	1	561 PYs	OR 2.33 (0.62–8.75)	2 vs. 4 mg: 0.23 (0.02–2.17)
	Upadacitinib	4	4	285 PYs	0	115 PYs	OR 1.77 (0.20–16.00)	15 vs. 30 mg: 4.36 (0.47–40)
	Filgotinib	1	0	178 PYs	0	42 PYs	–	–
	Peficitinib	3	0	179 PYs	0	42 PYs	–	–
	Decernotinib	2	0	49 PYs	0	17 PYs	–	–
Xie et al. [65]	Tofacitinib	12 for RA	1	881 PYs	2	263 PYs	OR 0.06 (0.00–0.95)	(Dose dependency: OR) 10 vs. 5 mg: 1.47 (0.25–8.50)
Yates et al. [66]	Overall	18 for IMIDs (11 for RA)	12 (10)	1950 PYs (1601PYs)	4 (3)	709 PYs (625 PYs)	RR 0.68 (0.36–1.29) for IMIDs	RR 0.44 (0.28–0.70) for PE RR 0.59 (0.31–1.15) for DVT
	Tofacitinib	7 (3)	2 (1)	1069 (758)	3 (2)	122 (77)	–	–
	Baricitinib	2 (2)	3 (3)	234 (234)	0	107 (107)	–	–
	Upadacitinib	6 (5)	6 (6)	475 (450)	1 (1)	378 (352)	–	–
	Filgotinib	3 (1)	1 (0)	172 (159)	0	102 (89)	–	–
Olivera et al. [67]	Overall	10 for IMIDs (6 for RA)	12 (11)	**n** = 3740 (2566)	3 (0)	**n** = 1403 (997)	RR 0.90 (0.32–2.54) for IMIDs	RR 1.70 (0.48–6.01) for RA
	Tofacitinib	4 (2)	3 (3)	2060 (1009)	3 (0)	536 (254)	–	–
	Baricitinib	1 (1)	2 (2)	374 (374)	0	210 (210)	–	–
	Upadacitinib	2 (2)	5 (5)	883 (883)	0	385 (385)	–	–
	Filgotinib	3 (1)	2 (1)	423 (300)	0	272 (148)	–	–
Bilal et al. [68]	Overall	25 for IMIDs (14 for RA)	50 (26)	**n** = 8933 (6254)	27 (4)	**n** = 3612 (2490)	OR 0.91 (0.57–1.47) for IMIDs	OR 1.11 (0.50–2.44) for RA
	Tofacitinib	7 (4)	5 (4)	3690 (2301)	5 (2)	908 (521)	OR 0.27 (0.08–0.89) for IMIDs	OR 0.54 (0.15–1.96) for 10 mg BID OR 0.49 (0.15–1.55) for 5 mg BID
	Baricitinib	5 (3)	9 (7)	1292 (862)	1 (1)	487 (348)	OR 1.12 (0.27–4.69) for IMIDs	OR 2.69 (0.42‒17.21) for 4 mg QD OR 3.05 (0.12‒75.43) for 2 mg QD
	Upadacitinib	4 (4)	12 (12)	2277 (2277)	1 (1)	1256 (1256)	OR 2.25 (0.55–9.25) for IMIDs	OR2.64 (0.27‒25.45) for 30 mg QD OR2.91 (0.69‒12.21) for 15 mg QD
	Filgotinib	2 (1)	2 (1)	358 (300)	0	206 (148)	OR 2.13 (0.22–20.64) for IMIDs	–
	Ruxolitinib	4 (0)	19 (0)	591 (0)	20 (0)	482 (0)	OR 0.85 (0.31–2.29) for IMIDs	–
	Decernotinib	2 (2)	2 (2)	514 (514)	0	217 (217)	OR 1.07 (0.18–6.43) for IMIDs	–
	Abrocitinib	1 (0)	1 (0)	211 (0)	0	56 (0)	OR 0.81 (0.03–20.03) for IMIDs	‒
Gimenez Poderos et al. [69]	Tofacitinib	5 for IMIDs (2 for RA)	–	–	–	–	OR 0.29 (0.10–0.84) for all doses	OR 1.19 (0.12–11.69) for 3 mg BID OR 0.18 (0.02–1.60) for 5 mg BID OR 0.19 (0.04–0.91) for 10 mg BID OR 0.32 (0.01–8.05) for 15 mg BID
	Baricitinib	5 for IMIDs (4 for RA)	–	–	–	–	OR 3.39 (0.82–14.04) for all doses	OR 3.05 (0.12–75.43) for 2 mg QD OR 3.64 (0.59–22.46) for 4 mg QD OR 3.00 (0.12–76.49) for 7 mg QD
Khoo et al. [70] ^‡^	Overall	27 for IMIDs (21 for RA)	12 (10)	*n* = 8363 (7270)	3 (3)	*n* = 3314 (2858)	RD 0.000 (− 0.002–0.003)	–
	Tofacitinib	10 (8)	3 (3)	4178 (3705)	2 (2)	1251 (1095)	0.000 (− 0.003–0.003)	–
	Baricitinib	7 (6)	3 (2)	2176 (1967)	1 (1)	1354 (1249)	0.000 (− 0.003–0.004)	–
	Upadacitinib	2 (2)	2 (2)	469 (469)	0	106 (106)	0.005 (− 0.015–0.024)	–
	Filgotinib	2 (0)	1 (0)	123 (0)	0	124 (0)	0.005 (− 0.020–0.030)	–
	Peficitinib	1 (1)	0	238 (238)	0	51 (51)	0.000 (− 0.027–0.027)	–
	Decernotinib	2 (1)	1 (1)	451(163)	0	112 (41)	0.001 (− 0.016–0.019)	–
	Fostamatinib	3 (3)	2 (2)	728 (728)	0	316 (316)	0.003 (− 0.006–0.012)	–

VTE events included PE and DVT, occurring both individually and in combination

^*^The ORs, RRs, and RDs of VTE events in patients receiving JAK inhibitors were calculated compared with those receiving placebo

^†^The numbers in parentheses represent study numbers, PYs, event numbers, or patient numbers for RA patients

^‡^Only PE events were included

*JAK*, Janus kinase; *RA*, rheumatoid arthritis; *IMID*, immune-mediated inflammatory disease; *VTE*, venous thromboembolism; PE*,* pulmonary embolism; *DVT*, deep vein thrombosis; *PYs*, person-years; *OR*, odds ratio; *RR*, risk ratio; *RD*, risk difference; *95% CI*, 95% confidence interval; *BID*, twice a day; *QD*, once a day

### VTE events in postmarketing studies using real-world registries

There are six postmarketing studies using real-world registries of RA and other IMID patients receiving JAK inhibitors [59, 71–75]. In a disproportionality analysis of data extracted from the postmarketing FDA’s Adverse Event Reporting System (FAERS) from March 2017, no evidence for increased reporting rates for DVT or PE was identified across three FDA-approved JAK inhibitors, tofacitinib, tofacitinib extended-release, and ruxolitinib (reporting odds ratios [RORs] and empirical Bayesian geometric means < 1). However, this study showed that pulmonary arterial thrombosis (PT) may be a potential safety issue for tofacitinib, with an ROR of 2.46 (95% CI 1.55–3.91) [71].

In descriptive and disproportionality analysis of data extracted in April 2019 from the World Health Organization global database (VigiBase) of individual case safety reports for tofacitinib and baricitinib, patients with DVT or PT/PE were older and more often received prothrombotic medications or antithrombotic treatment, suggesting a preexisting thromboembolic risk/event. In Europe, tofacitinib was associated with elevated reporting for DVT (ROR 2.37, 95% CI 1.23–4.56) and PT/PE (ROR 2.38, 95% CI 1.45–3.89). Similar increased reporting for DVT and PT/PE was observed in baricitinib-treated patients (ROR 3.47, 95% CI 2.18–5.52; and ROR 3.44, 95% CI 2.43–4.88, respectively). In the USA, tofacitinib was associated with an increased reporting rate of PT (ROR 2.05, 95% CI 1.45–2.90), but no evidence for elevated reporting was identified for DVT or PE (ROR < 1). DVT or PT/PE cases were not reported in baricitinib-treated patients in the US [72].

In an observational cohort study using claims data from two databases, the crude IRs of VTE (per 100 patient-years) for tofacitinib and TNF inhibitors in RA patients were 0.60 and 0.34 in the Truven MarketScan database (2012–2016, 1910 tofacitinib initiators and 32,164 TNF-inhibitor initiators) and 1.12 and 0.92 in the Medicare Claims database (2012–2015, 995 tofacitinib initiators and 16,091 TNF-inhibitor initiators), respectively. The PS-adjusted HRs had no statistically significant differences in VTE risk between tofacitinib and TNF inhibitors in either database, with a pooled HR of 1.33 (95% CI 0.78–2.24) [73]. The IRs of VTE in these databases were higher compared with those in the tofacitinib development program for RA [59]. With the accumulation of additional data from more recent years in these two databases (the MarketScan database [2012–2018] and the Medicare database [2012–2017]) and the inclusion of a third database (the Optum Clinformatics database [2012–2019]), an updated analysis was conducted by the same research group. The crude IRs of VTE (per 100 patient-years) for tofacitinib and TNF inhibitors were 0.42 and 0.35 in MarketScan, 1.18 and 0.83 in Medicare, and 0.19 and 0.34 in Optum, respectively. PS-adjusted HRs showed no statistically significant differences in VTE risk between tofacitinib and TNF inhibitors in any database, with a pooled HR of 1.13 (95% CI 0.77–1.65) [74].

In a post-approval comparative safety study using the US Corrona RA Registry, an ongoing longitudinal clinical registry from November 2012 through July 2018 (1999 tofacitinib initiators and 8358 TNF-inhibitor initiators), the IRs of VTE per 100 patient-years were 0.29 in tofacitinib initiators (5 mg twice daily in most cases) and 0.33 in bDMARD initiators, which were numerically similar between tofacitinib initiators and bDMARD initiators [75]. The IRs of VTE were numerically similar between RA patients in the Corrona Registry and those in the tofacitinib development program [59].

A recent ongoing postmarketing safety surveillance trial, ORAL Surveillance (Study A39212233), which is evaluating the safety of tofacitinib versus TNF inhibitors among RA patients aged ≥ 50 years and with at least one cardiovascular risk factor, raised concerns of a higher incidence of PE and all-cause mortality in patients treated with tofacitinib 10 mg twice daily compared with tofacitinib 5 mg twice daily or TNF inhibitors. In an ad hoc safety analysis (data cutoff February 2019), the IRs per 100 person-years in the treatments with tofacitinib 5 mg twice daily, tofacitinib 10 mg twice daily, and TNF inhibitors were 0.30, 0.38, and 0.18 for DVT and 0.27, 0.54, and 0.09 for PE, respectively. Compared with TNF inhibitors, the HRs (95% CI) for DVT and PE were 1.66 (0.60–4.57) and 2.99 (0.81–11.06) with tofacitinib 5 mg twice daily and 2.13 (0.80–5.69) and 5.96 (1.75–20.33) with tofacitinib 10 mg twice daily, respectively. The IRs of thromboembolic events observed in the tofacitinib development program for RA patients with cardiovascular or VTE risk factors were broadly consistent with those observed in the ORAL Surveillance trial. However, the IR of PE was significantly greater in patients receiving tofacitinib 10 mg twice daily in the ORAL Surveillance trial [59].

## Unanswered questions

As summarized above, in the systematic reviews and meta-analyses of data from clinical trials, the evidence was not sufficient to support the increased risk of VTE events during RA treatment with JAK inhibitors. These studies are limited by the small number of events reported and the limited overall exposure. In addition, patients with substantial cardiovascular risk factors and comorbidities are often excluded from such clinical trials. The postmarketing ORAL Surveillance analysis reported a significantly higher incidence of PE and all-cause mortality in RA patients treated with tofacitinib 10 mg twice daily. The FDA and EMA recommend that JAK inhibitors be avoided in patients with known VTE risk factors if alternative therapies are available. The package inserts for all approved JAK inhibitor products contain a box warning regarding the increased VTE risk [50].

Nevertheless, it is not entirely clear whether JAK inhibitors have a direct causal role in thromboembolic events or whether this risk simply represents a higher background thromboembolic risk in patients with RA (attributable to RA itself or its comorbidities) [53, 54]. There is a close relationship between the inflammatory activity of a given cytokine and its role in thrombus formation. In animal models, anti-inflammatory treatment is effective for thrombus resolution and the reduction of vessel wall damage [32, 76]. The JAK–STAT pathway can transmit signals from a variety of cytokines that have pro- or anti-thrombotic activity as well as pro- or anti-inflammatory activity. If blocking the JAK-STAT pathway results in a reduction of a particular cytokine’s inflammatory activity, it should induce the inhibition of prothrombotic activity. The real-world clinical data indicated that this is not entirely the case, however [77]. Whether the thromboembolic complications may be a class effect or a different JAK inhibitor may carry distinct VTE risks, possibly related to the specificity of JAK inhibitor action, remains unanswered [54, 77].

## Risk management of VTE in RA patients

When making a therapeutic decision of whether or not to start a JAK inhibitor for RA patients who are refractory to biological DMARDs, clinicians should carefully consider the following risk factors that predispose them to VTE events.

1. RA disease activity. RA is an independent risk factor for VTE. Disease activity is significantly associated with an increased risk of VTE. Our PE case presented in this review had received four biological DMARDs over 10 years, but the disease activity was poorly controlled. After the commencement of baricitinib, the patient achieved low disease activity, but DVT/PE occurred.
2. Comorbidities. Approximately 40% of RA patients suffer from some type of extra-articular manifestations during the course of their disease. The respiratory system is one of the most frequent targets of extra-articular manifestations [78]. In addition, the number of elderly RA patients with cardiovascular risk factors is increasing. Older patients are at increased risk of VTE because of multiple comorbid conditions and pharmaceutical changes related to drug metabolism and excretion [63]. Chronic kidney disease (CKD) and non-alcoholic fatty liver disease (NAFLD) have also been seen more commonly in this patient population [79, 80]. The presence of non-alcoholic steatohepatitis (NASH), a progressive form of NAFLD, is reported to downregulate the cytochrome P450 (CYP) 3A4 enzyme in the liver [81]. Tofacitinib is primarily metabolized through the CYP3A4 enzyme and excreted via the kidneys. Baricitinib is metabolized not via the CYP system but via the kidneys [50]. Thus, the presence of CKD and NAFLD/NASH can contribute to the increased risk of VTE associated with these JAK inhibitors. Dose adjustment is recommended in patients with renal impairment and/or NAFLD/NASH.
3. VTE and cardiovascular risk factors. As listed in the “Risk factors for VTE” section, numerous transient and persistent risk factors that can provoke VTE have been reported. Additional risk factors to be considered when prescribing JAK inhibitors include increased age and traditional cardiovascular risk factors such as obesity, diabetes, hypertension, hyperlipidemia, and smoking. It is important to recognize that the predictive values of these factors are not equal. Clinicians should consider both the strength of individual risk factors and the cumulative weight of all risk factors for each patient [18, 20].
4. Patient education. When a patient complains of warmth or redness in the leg, dyspnea, chest pain, and/or syncope during treatment with JAK inhibitors, clinicians should suspect the development of VTE/PE and initiate a rapid diagnostic workup. Prior to the initiation of JAK inhibitors, we should inform each patient of the number and strength of his/her risk factors for VTE, and advise them to seek prompt medical help if they develop clinical signs and symptoms that suggest VTE/PE.

## Limitations

We performed a literature search to comprehensively collect and analyze all sources relating to the risk of VTE events in RA patients receiving or not receiving JAK kinase inhibitors. We obtained relevant data from a variety of articles published in rheumatology, pharmacology, cardiology, hematology, and epidemiology journals, which contributed to the reduction of a selection bias. In addition, we included detailed information on the massive and acute PE case that we experienced during baricitinib treatment for multiple biologic-resistant RA, which provides critical information regarding the risk management of VTE events in RA patients who are scheduled to receive JAK inhibitor therapy.

There are several limitations to this study. First, we undertook literature searches solely through the Medline database, and, therefore, we might have missed some relevant studies. Second, we mainly focused on VTE events associated with the five JAK inhibitors approved for RA, namely, tofacitinib, baricitinib, upadacitinib, filgotinib, and peficitinib. Several new JAK inhibitors have been developed for IMIDs, but detailed data on VTE risk of individual new-generation JAK inhibitors were not available in the literature. Third, our review focused on the VTE risk in RA patients, and did not cover patients with other IMIDs such as psoriasis, inflammatory bowel diseases, and other inflammatory rheumatic diseases. We cannot entirely exclude the possibility that there may be a difference in VTE risk between patients with RA and those with non-RA IMIDs.

## Conclusions

To date, the evidence is limited and insufficient to support the idea that there is an increased risk of VTE during RA treatment with JAK inhibitors. In addition, the exact mechanisms of how JAK inhibitors might increase the risk of VTE remain to be clarified. A signal of VTE/PE risk with JAK inhibitors has been noted in RA patients who are already at high risk, however. Clinicians should follow the regulatory recommendations to avoid the use of JAK inhibitors in patients with cardiovascular and VTE risk factors if alternative therapies are available. If suitable alternatives are not available, clinicians should prescribe JAK inhibitors with caution, taking the number and strength of VTE risk factors for each RA patient into careful consideration.

## Declarations

### Publishing agency

We did not use the services of external publishing agents.

### Disclaimer

No part of this manuscript has been copied or published elsewhere.
